# Effect of prehabilitation for patients undergoing gastric cancer surgery: a protocol of a systematic review and meta-analysis

**DOI:** 10.1136/bmjopen-2024-083914

**Published:** 2024-09-10

**Authors:** Linyu Xu, Xutong Zheng, Yaxi Yan, Bei Wang, Aiping Wang

**Affiliations:** 1 Department of Public Service, The First Affiliated Hospital of China Medical University, Shenyang, Liaoning Province, China; 2 China Medical University, Shenyang, Liaoning Province, China

**Keywords:** Meta-Analysis, GASTROENTEROLOGY, Gastrointestinal tumours

## Abstract

**Abstract:**

**Introduction:**

Gastric cancer is a high-risk cancer with surgical treatments often leading to significant postoperative complications and mortality. Prehabilitation, involving exercise, nutrition and psychological support before surgery, aims to boost patients’ physical and mental health. While effective in other cancers, its benefits for gastric cancer need further study. This research will evaluate the impact of trimodal prehabilitation on patient outcomes in gastric cancer surgery, aiming to reduce complications and expedite recovery.

**Methods and analysis:**

This study will systematically review randomised controlled trials and cohort studies evaluating the role of prehabilitation in people undergoing gastric cancer resection. The primary outcomes of interest will include overall postoperative complications and length of hospital stay. The secondary outcomes of interest will include mortality, readmission rate or functional recovery. Databases including PubMed, EMBASE, CINAHL, CENTRAL, Chinese BioMedical Literature Database (CBM), Chinese National Knowledge Infrastructure (CNKI), Wanfang database and Chinese Science and Technology Periodicals (VIP) will be searched. All studies will be screened and selected using the criteria described in ‘population, intervention/exposure, comparison, outcome and study design’ format. Two independent reviewers will screen studies for relevance and methodological validity. Data from included studies will be extracted through a customised, preset data extraction sheet. The Cochrane Review Manager (V.5.3, Nordic Cochrane Centre, Copenhagen, Denmark) software will be used to perform the meta-analysis.

**Ethics and dissemination:**

Ethics approval is not required for this study as all results will be based on published papers. No primary data collection will be needed. Study findings will be presented at scientific conferences or published in a peer-reviewed scientific journal.

**PROSPERO registration number:**

CRD42023488469.

**STRENGTHS AND LIMITATIONS OF THIS STUDY**

- The role of prehabilitation in people undergoing gastric cancer resection will be estimated by meta-analysing outcomes data and the quality of the body of literature will be evaluated using Grading of Recommendation, Assessment, Development and Evaluation.
- Variations in surgical procedures and prehabilitation interventions/exposures may preclude meta-analysis and affect the overall level of evidence.
- The scarcity of randomised controlled trials and cohort studies may be the main limitation of the study.

## Introduction

 According to the latest report of Global Cancer Statistics 2020 (GLOBOCAN 2020), gastric cancer is the fifth most commonly diagnosed and the fourth most life-threatening cancer.^1^ Surgery, with or without neoadjuvant therapy, remains the primary curative treatment modality for most gastric cancer patients.^2^ However, despite the continuous advancements in modern medicine, surgical treatments for gastric cancer still carry a relatively high risk of postoperative complications and mortality. According to a relevant study, perioperative complications can impact outcomes following gastrectomy for cancer, with mortality and morbidity rates reaching as high as 10%–40%.^3^ Current evidence indicates that neoadjuvant chemotherapy is a beneficial therapeutic measure.^4^,^6^ Although preoperative cytotoxic treatment enhances oncological results, it adversely affects patients’ physical and nutritional well-being, contributes to sarcopenia and diminishes physiological reserves, consequently amplifying the risks associated with surgery.^7^,^9^ Therefore, a new strategy needs to be adopted to improve outcomes for gastric cancer surgery patients.

Recently, a new preoperative management strategy, termed prehabilitation, has been proposed within the framework of enhanced recovery after surgery (ERAS).^10^ In contrast to ERAS, which primarily focuses on the postoperative period, prehabilitation aims to enhance the organism’s baseline level through preoperative rehabilitation. This is intended to bolster the patient’s ability to withstand surgical stress and expedite the postoperative recovery process.

Prehabilitation programmes were initially developed with preoperative physical exercise as the core intervention to improve a patient’s functional capacity reserve.^10^,^12^ With a deepening comprehension of the influence of preoperative nutrition on postoperative recovery, the purview of prehabilitation progressively extended into the domain of nutritional considerations.^10 13 14^ Subsequently, the integration of psychosocial support into prehabilitation protocols emerged as a pivotal constituent.^10^ Therefore, the trimodal prehabilitation plan includes the preoperative components of exercise, nutrition and psychosocial support.^10 15 16^ Additionally, some studies categorise the components of prehabilitation into one (unimodal) or several (multimodal) interventions.

The outcome measures used to assess the effectiveness of prehabilitation can be categorised into three types: preoperative outcomes, short-term postoperative outcomes and long-term postoperative outcomes.^17^ These include overall postoperative complications, mortality, readmission rate, functional recovery and length of hospital stay (LOS).^18^,^21^ Presently, a range of randomised controlled trials (RCTs) in the surgical field has substantiated the effectiveness of prehabilitation, and a plethora of systematic reviews has been published stemming from these studies, encompassing diverse areas including breast,^22^ cardiothoracic,^23^ oesophagogastric,^24^ colorectal,^25 26^ pancreatic,^27^ prostate^28^ and orthopaedic.^29^ So far, several studies already investigated the role of prehabilitation in oesophagogastric surgery. Zhao *et al*^24^ conducted a systematic review and meta-analysis on the effectiveness of unimodal or multimodal prehabilitation on patients undergoing surgery for oesophagogastric cancer and reported slight improvement in prehabilitation reduced the incidence of all complications (risk ratio, RR 0.79, 95% CI 0.66 to 0.93, p=0.006), pulmonary complications (RR 0.61, 95% CI 0.47 to 0.79, p=0.0002) and severe complications (RR 0.63, 95% CI 0.47 to 0.84, p=0.002) and shortened the length of stay (mean difference, MD=−1.92, 95% CI −3.11 to −0.73, p=0.002) compared with usual care. However, most of these studies focused on patients with oesophageal cancer, with limited data available for gastric cancer.^24 30^

Currently, there is still significant heterogeneity in the composition of prehabilitation programmes. Furthermore, up to now, there has been no research investigating the effectiveness of trimodal prehabilitation for patients with gastric cancer. This systematic review and meta-analysis aim to comprehensively delineate the constituents of prehabilitation plans for patients with gastric cancer undergoing surgery within the trimodal prehabilitation model while evaluating the anticipated impact of prehabilitation on postoperative outcomes.

### Objectives

In this paper, we report on the protocol of a systematic review and meta-analysis that will:

- Synthesise the available evidence on the role of prehabilitation in patients undergoing gastric cancer surgery.
- Summarise the composition of prehabilitation programmes for patients undergoing gastric cancer surgery.
- Evaluate whether prehabilitation has an impact on postoperative outcomes in patients with gastric cancer.

## Methods

### Study registration

The protocol is developed in line with the guidelines of Preferred Reporting Items for Systematic Reviews and Meta-analyses Protocols (PRISMA).^31^ The final systematic review will be conducted in line with the PRISMA statement,^32^ and the guidance of the Cochrane Handbook for Systematic Reviews of Interventions.^33^ The project began in November 2023 and is expected to be completed by March 2024.

### Patient and public involvement

Patients and/or the public were not involved in the design, or conduct, or reporting, or dissemination plans of this research.

### Eligibility criteria

Studies will be included in the final review if they meet the following inclusion criteria:

- Types of studies: RCTs or cohort studies reported in English or Chinese published as full-text manuscripts will be included. Descriptive study designs, protocol manuscript, reviews, editorials, case reports, conference proceedings, abstracts, dissertations and book chapters will be excluded.
- Types of participants: Patients (≥18 years) who are undergoing gastric cancer surgery, with no restriction on race and gender. Patients with metastases will be excluded, as this may result in confounders of the stage of disease affecting the postoperative outcomes.
- Types of interventions/exposure and comparators: The intervention/exposure group could be exercise, nutritional optimisation or psychological support in combination as defined by original studies. The control group could be routine care, usual care, waitlist control, standard treatment or conventional care interventions/exposure.
- Types of outcome measures: The primary outcomes of interest will include overall postoperative complications and LOS. The secondary outcomes of interest will include mortality, readmission rate or functional recovery.

### Search strategy

The following key electronic bibliographic databases will be searched systematically from inception to January 2024:

- PubMed.
- EMBASE.
- CINAHL.
- Cochrane Central Register of Controlled Trials (CENTRAL).
- Chinese Biomedical Literature Database (CBM).
- China National Knowledge Infrastructure (CNKI).
- WANFANG database.
- Chinese Scientific Journal Database (VIP).

To construct the search, the Patient, Intervention/Exposure, Comparison, Outcome, Study scheme will be used, the search strategy will search for ‘gastric cancer’ AND ‘prehabilitation/exercise/nutritional/psychological’. For each of the ‘patient’ ‘intervention/exposure’, and ‘study design’ concept, we will combine synonyms and MeSH terms with the ‘OR’ operator. The initial proposed search strategy can be found in online supplemental appendix 1. This strategy will be adapted for use in the other databases. In addition, we will handsearch the reference lists of all the included trials, relevant reviews and publications on the topic, to identify any potentially eligible studies.

### Study selection

**Figure 1:**
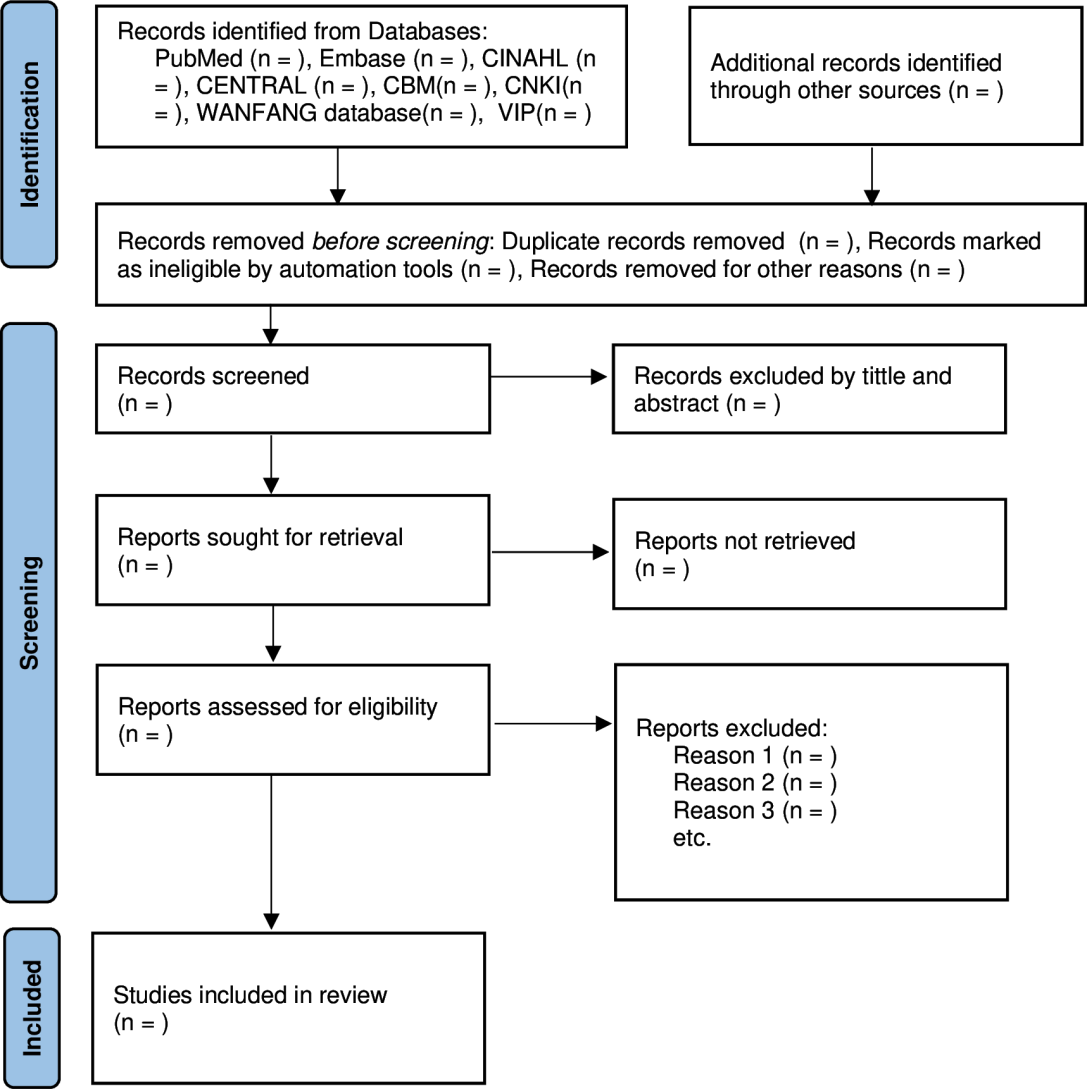
Flow chart of the study selection procedure: PRISMA 2020 flow diagram for new systematic reviews which included searches of databases and registers only. PRISMA, Preferred Reporting Items for Systematic Reviews and Meta-Analyses.

The specific bibliographic software EndNote V.20 will be used to store, organise and manage data. The retrieved publications will be imported into EndNote and any duplicates will be identified and removed. Two reviewers (LY and YX) will independently screen the titles, abstracts and keywords of relevant studies for their eligibility according to the predefined criteria. After preliminary screening, the remaining studies will subsequently undergo a detailed full-text review, and the explicit reasons for the exclusion of excluded studies will be recorded. Disagreements will be solved by discussion or consultation with a third reviewer (WB). The study selection procedure will be depicted in the PRISMA flow diagram (see figure 1).^31^

### Data extraction and management

Two reviewers (LY and YX) will independently extract the following data from the identified studies, and any disagreements will be solved by discussion or consultation with a third author (WB):

- Publication details: title, authors, publication year, country /region of study, journal.
- Study characteristics: study design, method of randomisation, allocation concealment, baseline comparability of groups, method and object of blinding, completeness of outcome data.
- Participants: age, sample characteristics, sample size.
- Interventions/exposures: duration, location (community, home-based, hospital), method of prehabilitation delivery (home, face to face, electronic means), components of the prehabilitation programme (exercise, nutritional and/or psychological).
- Outcomes: primary and secondary outcomes. Health-related quality of life and functional walking capacity will also be reported as appropriate.

### Risk of bias assessment

Two reviewers (LY and YX) will independently assess the risk of bias for the identified studies. The Cochrane Risk of Bias 2 tool will be used to assess the risk of bias in the randomised controlled studies. Using this tool, we will assess studies based on several criteria: random sequence generation, blinding of outcome assessment, blinding of participants and personnel, selective reporting, incomplete outcome and other bias.^34^ Disagreements between reviewers will be resolved by consensus or through consultation with a third reviewers (WB). The level of bias (high, low and unclear) will be graded for each important outcome and study and then be presented graphically in a table.

### Data analysis and synthesis

If sufficient data are available to evaluate a common outcome and the studies are comparable with regard to design, methodology and intervention/exposure, a meta-analysis will be conducted to acquire estimates of the pooled effect. In case there are limited data available for an outcome, we will provide a narrative summary of the individual studies’ findings instead. RevMan V.5.3 software will be used for meta-analysis.

We will calculate RR and its 95% CI for binary outcomes, whereas continuous data will be expressed as group post-test means and SDs to calculate effect sizes. We will communicate effect sizes preferentially in the form of MDs and 95% CIs, but when different scales were used to measure the same outcome, we will calculate standardised MDs instead, with corresponding 95% CIs.

Thresholds for the interpretation of the I^2^ statistic can be misleading, it is recommended that I^2^ values of 0%–40%, 30%–60%, 50%–90% or 75%–100% be interpreted as indicating not-important, moderate, substantial or considerable heterogeneity.^35^ Due to the variations of complex interventions, the random-effects model will be used to measure effect sizes.^36^ When sufficient data are available, analyses will be conducted to explore factors that explain heterogeneity, including subgroup analyses based on surgical methods, population and interventions/exposures. If the data permit, sensitivity analyses will be performed to assess the impact of each individual study on the overall meta-analysis summary estimates.

### Meta-biases

The studies will be assessed for publication bias and selective reporting of outcomes. When insufficient data are available, attempts will be made to contact the authors for further information and will be reported clearly. We will also scrutinise all eligible studies as to whether intended outcome measures were actually reported in the studies. In the case there are 10 or more studies assessing 1 common outcome, funnel plots will be generated to assess publication bias.

### Confidence in cumulative evidence

We will assess the certainty of evidence using the Grading of Recommendations Assessment, Development and Evaluation system.^37 38^ The results will be categorised as high, moderate, low and very low certainty of evidence. The certainty can be downgraded for five reasons: risk of bias, imprecision, inconsistency, indirectness and publication bias.

## Ethics and dissemination

Ethics approval is not required for this study as only data from published papers will be used. The findings from this systematic review and meta-analysis will be submitted for publication in a peer-reviewed scientific journal and for presentation at academic conferences.

## supplementary material

10.1136/bmjopen-2024-083914 online supplemental appendix 1
