# Supplementary material for: Effect of prehabilitation for patients undergoing gastric cancer surgery: a protocol of a systematic review and meta-analysis
Source: BMJ Open. 2024 Sep 10;14(9):e083914. doi: 10.1136/bmjopen-2024-083914 (PMC11409279; doi:10.1136/bmjopen-2024-083914)
Supplement: online supplemental appendix 1 [file bmjopen-14-9-s001.pdf]

Appendix 1
Search Strategy:

PubMed

#	Searches
1	"gastric neoplas*"[Title/Abstract] OR "gastric cancer*"[Title/Abstract] OR "stomach neoplas*"[Title/Abstract] OR "stomach cancer*"[Title/Abstract] OR "stomach neoplasms"[MeSH Terms]
2	"perioperative care"[MeSH Terms] OR "perioperative"[Text Word] OR "peri-operative"[Text Word] OR "preoperative care"[MeSH Terms] OR "pre-operative"[Text Word] OR "preoperative"[Text Word] OR "prehab*"[Text Word] OR "pre hab*"[Text Word]
3	"exercise"[MeSH Terms] OR "exercise*"[Text Word] OR "training"[Text Word] OR "physical*"[Text Word] OR "physiological*"[Text Word] OR "physiotherapy"[Text Word]
4	"nutritional support"[MeSH Terms] OR "nutrition therapy"[MeSH Terms] OR "diet"[MeSH Terms] OR "nutri*"[Text Word] OR "diet*"[Text Word] OR "supplement*"[Text Word]
5	"psychological*"[Text Word] OR "psychotherapy"[Text Word] OR "cognitive therapy"[Text Word]
6	3 OR 4 OR 5
7	1 AND 2 AND 6

EMBASE

#	Searches
1	'gastric neoplas*':ab,ti OR 'gastric cancer*':ab,ti OR 'stomach neoplas*':ab,ti OR 'stomach cancer*':ab,ti OR 'stomach neoplasm'/exp
2	'perioperative care'/exp OR 'perioperative':ab,ti OR 'peri-operative':ab,ti OR 'preoperative care'/exp OR 'pre-operative':ab,ti OR 'preoperative':ab,ti OR 'prehab*':ab,ti OR 'pre hab*':ab,ti
3	'exercise'/exp OR 'exercise*':ab,ti OR 'training':ab,ti OR 'physical*':ab,ti OR 'physiological*':ab,ti OR 'physiotherapy':ab,ti
4	'nutritional support'/exp OR 'nutrition therapy'/exp OR 'diet'/exp OR 'nutri*':ab,ti OR 'diet*':ab,ti OR 'supplement*':ab,ti
5	'psychological*':ab,ti OR 'psychotherapy':ab,ti OR 'cognitive therapy':ab,ti
6	3 OR 4 OR 5
7	1 AND 2 AND 6 AND [article]/lim AND [english]/lim AND [embase]/lim

CINAHL

S	Searches
1	'(MH "Stomach Neoplasms") OR TI "gastric neoplas*" OR TI "gastric cancer*" OR TI "stomach neoplas*" OR TI "stomach cancer*"
2	(MH "Perioperative Care") OR TI "perioperative" OR TI "peri-operative" OR (MH "Preoperative Care") OR TI "preoperative" OR TI "pre-operative" OR TI

	"prehab*" OR TI "pre hab*"
3	(MH "Exercise") OR TI "exercise*" OR TI "training" OR TI "physical*" OR TI "physiological*" OR TI "physiotherapy"
4	(MH "Nutritional Support") OR (MH "Nutrition Therapy") OR (MH "Diet") OR TI "nutri*" OR TI "diet*" OR TI "supplement*"
5	TI "psychological*" OR TI "psychotherapy" OR TI "cognitive therapy"
6	3 OR 4 OR 5
7	1 AND 2 AND 6

CENTRAL

#	Searches
1	MeSH descriptor: [Stomach Neoplasms] explode all trees
2	(gastric neoplas*):ti,ab,kw OR (gastric cancer*):ti,ab,kw OR (stomach neoplas*):ti,ab,kw OR (stomach cancer*):ti,ab,kw
3	1 OR 2
4	MeSH descriptor: [Perioperative Care] explode all trees
5	MeSH descriptor: [Preoperative Care] explode all trees
6	(perioperative):ti,ab,kw OR (peri-operative):ti,ab,kw OR (pre-operative):ti,ab,kw OR (preoperative):ti,ab,kw OR (prehab*):ti,ab,kw OR (pre hab*):ti,ab,kw
7	4 OR 5 OR 6
8	MeSH descriptor: [Exercise] explode all trees
9	(exercise*):ti,ab,kw OR (training):ti,ab,kw OR (physical*):ti,ab,kw OR (physiological*):ti,ab,kw OR (physiotherapy):ti,ab,kw
10	8 OR 9
11	MeSH descriptor: [Nutritional Support] explode all trees
12	MeSH descriptor: [Nutrition Therapy] explode all trees
13	MeSH descriptor: [Diet] explode all trees
14	(nutri*):ti,ab,kw OR (diet*):ti,ab,kw OR (supplement*):ti,ab,kw
15	11 OR 12 OR 13 OR 14
16	(psychological*):ti,ab,kw OR (psychotherapy):ti,ab,kw OR (cognitive therapy):ti,ab,kw
17	10 OR 15 OR 16
18	3 AND 7 AND 17 in Trials

Chinese Biomedical Literature Database (CBM)

#	Searches
	("gastric cancer"[common fields: intelligent] OR "stomach neoplasms"[common fields: intelligent]) AND ("exercise"[common fields: intelligent] OR "nutrition"[common fields: intelligent] OR "psychological"[common fields: intelligent]) AND "preoperative"[common fields: intelligent]<br>("胃癌"[核心字段:智能] OR "胃肿瘤"[核心字段:智能]) AND ("运动"[核心字段:智能] OR "营养"[核心字段:智能] OR "心理"[核心字段:智能]) AND "术前"[核心字段:智能]

The Chinese National Knowledge Infrastructure (CNKI)

#	Searches
	("gastric cancer"[Title/Abstract] AND "stomach neoplasms"[Title/Abstract]) AND ("exercise"[Title/Abstract] OR "nutrition"[Title/Abstract] OR "psychological"[Title/Abstract]) AND "preoperative"[Title/Abstract]<br>（篇关摘：胃癌 + 胃肿瘤(精确)）AND（篇关摘：运动 + 营养 + 心理(精确)）AND（篇关摘：术前(精确)）

WANFANG database

#	Searches
	Title or Keywords: (gastric cancer OR stomach neoplasms) AND Title or Keywords: (preoperative) AND Title or Keywords: (exercise OR nutrition OR psychological)<br>题名或关键词:(胃癌 or 胃肿瘤) and 题名或关键词:(术前) and 题名或关键词:(运动 or 营养 or 心理)

Chinese Scientific Journal Database (VIP)

#	Searches
	("gastric cancer"[Title/Abstract] AND "stomach neoplasms"[Title/Abstract]) AND ("exercise"[Title/Abstract] OR "nutrition"[Title/Abstract] OR "psychological"[Title/Abstract]) AND "preoperative"[Title/Abstract]<br>（篇关摘：胃癌 + 胃肿瘤(精确)）AND（篇关摘：运动 + 营养 + 心理(精确)）AND（篇关摘：术前(精确)）
