# Supplementary material for: Effect of prehabilitation for patients undergoing gastric cancer surgery: a protocol of a systematic review and meta-analysis
Source: BMJ Open. 2024 Sep 10;14(9):e083914. doi: 10.1136/bmjopen-2024-083914 (PMC11409279; doi:10.1136/bmjopen-2024-083914)
Supplement: Supplementary file 2 [file bmjopen-2024-083914.reviewer_comments.pdf]

PEER REVIEW HISTORY

BMJ Open publishes all reviews undertaken for accepted manuscripts. Reviewers are asked to complete a checklist review form and are provided with free text boxes to elaborate on their assessment. These free text comments are reproduced below.

ARTICLE DETAILS

**TITLE (PROVISIONAL)**	Effect of prehabilitation for patients undergoing gastric cancer surgery：a protocol of a systematic review and meta-analysis
**AUTHORS**	Xu, Linyu; Zheng, Xutong; Yan, Yaxi; Wang, Bei; Wang, Aiping

VERSION 1 - REVIEW

**REVIEWER NAME**	Li, Peng
**REVIEWER AFFILIATION**	Zhejiang Provincial People's Hospital (Affiliated People's Hospital), Hangzhou Medical College, Cancer Center, Department of Gastroenterology
**REVIEWER CONFLICT OF INTEREST**	There are no competing interests.
**DATE REVIEW RETURNED**	20-Jan-2024

**GENERAL COMMENTS**	The manuscript 'Effect of prehabilitation for patients undergoing gastric cancer surgery: a protocol of a systematic review and meta-analysis' is a well-prepared and well-written protocol. There are some minor issues that need to be considered before acceptance:<br><br>1. Prehabilitation is an important aspect of perioperative management for gastric cancer surgery. But some heterogeneity might affect outcomes when conducting the systematic review and meta-analysis, such as surgical methods, tumor staging.<br>2. Available sensitivity analysis and subgroup analysis are needed to reduce heterogeneity.<br>3. In the part of Eligibility criteria, 'Types of participants: patients (≥18 years) who are undergoing gastric surgery,…'. should be 'undergoing gastric cancer surgery'.

**REVIEWER NAME**	Du, Jing
**REVIEWER AFFILIATION**	Zhejiang Provincial People's Hospital (Affiliated People's Hospital), Hangzhou Medical College, Cancer Center, Department of Gastroenterology
**REVIEWER CONFLICT OF INTEREST**	No
**DATE REVIEW RETURNED**	06-Feb-2024

**GENERAL COMMENTS**	In eligibility criteria, the authors claim that 'types of participants: patients (≥18 years) who are undergoing gastric surgery, with no restriction on age, race and gender'. 'Age over 18' and 'no restriction on age' are contradictory.<br>The authors extract data only from randomised controlled trials (RCTs), cohort study data could be included for consideration, too.

**REVIEWER NAME**	Koh, Frederick Hong Xiang
**REVIEWER AFFILIATION**	Sengkang General Hospital
**REVIEWER CONFLICT OF INTEREST**	None
**DATE REVIEW RETURNED**	27-May-2024

**GENERAL COMMENTS**	thank you for the opportunity to review this piece of work. This is a protocol paper for a systematic review and metaanalysis looking into patients with gastric cancer and evaluating whether prehabilitation has an impact on “post operative outcomes”.<br><br>This a relevant piece of work as there is increasing trend to incorporate rehabilitation into perioperative management of surgical patients and there is an increasing trend to suggest that multimodal prehabiltation may be the way to go to help optimise patients better for their major operation.<br><br>This protocol has checked most methodological boxes in running a metaanalysis and clearly the authors are experienced in this area.<br><br>This protocol has, however, still some methodological flaws which can be refined before consideration of publication:<br><br>1. Having only RCTs included might severely limit the number of studies included, and as literature is moving towards real-world data, well designed cohort studies for rehabilitation’s should be considered to offer a pool effect of prehabilitation that is closer to reality than just having an artificial study population that we get in RCTs. Whilst this may reduce the “grade of evidence” of the manuscript, it would certainly offer a more comprehensive review of the topic for gastric cancer patients.<br>2. for the study population, would the authors consider including curative gastric cancer patients rather than those with metastases? as this may result in confounders of stage of disease affecting the postoperative outcomes such as overall survival and how well they do post surgery (objective clinical measures and Quality of life metrics)<br>3. the outcome is not well articulated yet in the abstract whilst it is mentioned in the full text.<br>4. In terms of intervention, one of the biggest problems of trials/studys/metaanalysis of prehabilitation studies are that the inclusion of hetergenous interventions which are poorly defined. this amalgamation of hetergeneous studies have resulted in conflicting aggregated results which has swung the outcomes of prehabilitation. Prehabilitation should be taken in the same vein as ERAS, where an accumulation of marginal gains through multiple interventions led to a bigger benefit to improving the patients’ baseline physiological state to withstand the surgical stress. And thus, i urge the authors to consider only studies which performed multimodal prehabilitation which include nutrition, exercise and either anxiety control / smoking cessation / inspiratory muscle training / etc. In this way, true effects and impact of multimodal prehabilitation on surgical outcomes can be measured and not get diluted with lesser interventions.<br>5. for the analysis of data, only random effects model should be used in fact, and a prediction window should be stated in order to determine heterogeneity rather than I2 if possible. this is a common

	misconception of statistics being used to determine the type of pooling analysis to be performed.<br>6. Sensitivity analysis to check for validity of the data should be mentioned to be done at least for the primary outcomes and all statistically significant outcomes in order to ensure validity of the data presented. methods of sensitivity analysis should be mentioned too. most conventionally, a leave-one-out analysis (also termed as bootstrapping) can be done and provided in the supplementary tables.

VERSION 1 – AUTHOR RESPONSE

Reviewer #1：The manuscript 'Effect of prehabilitation for patients undergoing gastric cancer surgery: a protocol of a systematic review and meta-analysis' is a well-prepared and well-written protocol. There are some minor issues that need to be considered before acceptance:

Comment 1: Prehabilitation is an important aspect of perioperative management for gastric cancer surgery. But some heterogeneity might affect outcomes when conducting the systematic review and meta-analysis, such as surgical methods, tumor staging.

Response: Thank you for your valuable comment. We have made the additions according to your suggestions (Page 10).

Comment 2: Available sensitivity analysis and subgroup analysis are needed to reduce heterogeneity.

Response: Thank you for your valuable comment. We have made the additions according to your suggestions (Page 10).

Comment 3: In the part of Eligibility criteria, 'Types of participants: patients (≥18 years) who are undergoing gastric surgery,…'. should be 'undergoing gastric cancer surgery'.

Response: Thank you for your valuable comment. We are very sorry for this oversight and have made the necessary corrections (Page 6).

Reviewer #2：

Comment 1: In eligibility criteria, the authors claim that 'types of participants: patients (≥18 years) who are undergoing gastric surgery, with no restriction on age, race and gender'. 'Age over 18' and 'no restriction on age' are contradictory.

Response: We apologize for the contradiction caused by our carelessness. The errors in the article have been reviewed and corrected. We appreciate your elaborate efforts in reviewing. (Page 6)

Comment 2: The authors extract data only from randomised controlled trials (RCTs), cohort study data could be included for consideration, too.

Response: We agree with your insightful points. In response, we have revised both the abstract and methods sections of our manuscript to include cohort studies in addition to RCTs.

Reviewer #3：thank you for the opportunity to review this piece of work. This is a protocol paper for a systematic review and metaanalysis looking into patients with gastric cancer and evaluating whether prehabilitation has an impact on “post operative outcomes”.

This a relevant piece of work as there is increasing trend to incorporate rehabilitation into perioperative management of surgical patients and there is an increasing trend to suggest that multimodal prehabiltation may be the way to go to help optimise patients better for their major operation.

This protocol has checked most methodological boxes in running a metaanalysis and clearly the authors are experienced in this area. This protocol has, however, still some methodological flaws which can be refined before consideration of publication:

Comment 1: Having only RCTs included might severely limit the number of studies included, and as literature is moving towards real-world data, well designed cohort studies for rehabilitation’s should be considered to offer a pool effect of prehabilitation that is closer to reality than just having an artificial study population that we get in RCTs. Whilst this may reduce the “grade of evidence” of the manuscript, it would certainly offer a more comprehensive review of the topic for gastric cancer patients.

Response: We agree with your insightful points. In response, we have revised both the abstract and methods sections of our manuscript to include cohort studies in addition to RCTs.

Comment 2: for the study population, would the authors consider including curative gastric cancer patients rather than those with metastases? as this may result in confounders of stage of disease affecting the postoperative outcomes such as overall survival and how well they do post surgery (objective clinical measures and Quality of life metrics)

Response: Thank you for your valuable comment. Following your advice, we have revised the Eligibility Criteria in the Methods section (Page 6). Additionally, we have included your statement that 'as this may result in confounders of stage of disease affecting the postoperative outcomes' into our manuscript.

Comment 3: the outcome is not well articulated yet in the abstract whilst it is mentioned in the full text.

Response: Thank you for your valuable comment. We have revised the abstract to clearly articulate the outcome, ensuring it is consistent with the full text. We appreciate your attention to this detail and your valuable input in helping us improve our manuscript. (Page 2)

Comment 4: In terms of intervention, one of the biggest problems of trials/studys/metaanalysis of prehabilitation studies are that the inclusion of hetergenous interventions which are poorly defined. this amalgamation of hetergeneous studies have resulted in conflicting aggregated results which has swung the outcomes of prehabilitation. Prehabilitation should be taken in the same vein as ERAS, where an accumulation of marginal gains through multiple interventions led to a bigger benefit to improving the patients' baseline physiological state to withstand the surgical stress. And thus, i urge the authors to consider only studies which performed multimodal prehabilitation which include nutrition, exercise and either anxiety control / smoking cessation / inspiratory muscle training / etc. In this way, true effects and impact of multimodal prehabilitation on surgical outcomes can be measured and not get diluted with lesser interventions.

Response: Your suggestion is valuable and meaningful. Relevant study(Enhanced Recovery After Surgery 2020) also indicate that a single variable cannot accurately and reliably reflect functional status indirectly. Therefore, it is recommended to establish a solid rational foundation that emphasizes the synergistic effect of exercise, nutrition, and mental health. Following your advice, we have revised the Eligibility Criteria in the Methods section on page six to consider only studies that performed multimodal prehabilitation. (Page 6)

Comment 5: for the analysis of data, only random effects model should be used in fact, and a prediction window should be stated in order to determine heterogeneity rather than I2 if possible. this is a common misconception of statistics being used to determine the type of pooling analysis to be performed.

Response: Your suggestion is valuable and meaningful. We have revised in the third paragraph of the Data Analysis and Synthesis section (Page 6). Based on your comments, we have read and learned some papers, such as(Borenstein et al. 2010; Dettori, Norvell, and Chapman 2022). The strategy of starting with a fixed-effect model and then moving to a random-effects model if the test for heterogeneity is significant relies on a flawed logic and should be strongly discouraged. The selection of a model should be based solely on the question of which model fits the distribution of effect sizes and thus takes account of the relevant source(s) of error. When studies are gathered from the published literature, the random-effects model is generally a more plausible match. The fixed-effect model is based on the assumption that all studies in the meta-analysis share a common (true) effect size. Under the fixed-effect model, the summary effect is an estimate of the effect which is common to all studies in the analysis. Under the random-effects model, the summary effect is an estimate of the mean of a distribution of true effects. Based on your comments, our thinking has become much clearer. Thank you for your meticulous effort during the review process.

Comment 6: Sensitivity analysis to check for validity of the data should be mentioned to be done at least for the primary outcomes and all statistically significant outcomes in order to ensure validity of the data presented. methods of sensitivity analysis should be mentioned too. most conventionally, a leave-one-out analysis (also termed as bootstrapping) can be done and provided in the supplementary tables.

Response: Thank you for your valuable comment. We have made the additions according to your suggestions (Page 10).

Special thanks to you for your valuable comments.

We sincerely appreciate the efforts of the Editors and Reviewers, and hope that the corrections will meet with your approval.

Once again, thank you very much for your comments and suggestions.

References:

Borenstein, M., L. V. Hedges, J. P. Higgins, and H. R. Rothstein. 2010. 'A basic introduction to fixed-effect and random-effects models for meta-analysis', Res Synth Methods, 1: 97-111.

Dettori, J. R., D. C. Norvell, and J. R. Chapman. 2022. 'Fixed-Effect vs Random-Effects Models for Meta-Analysis: 3 Points to Consider', Global Spine J, 12: 1624-26.

Enhanced Recovery After Surgery. 2020. (Springer Cham).

VERSION 2 – REVIEW

**REVIEWER NAME**	Koh, Frederick Hong Xiang
**REVIEWER AFFILIATION**	Sengkang General Hospital
**REVIEWER CONFLICT OF INTEREST**	None.
**DATE REVIEW RETURNED**	18-Jul-2024

**GENERAL COMMENTS**	This is a revision of an earlier submitted protocol paper.<br><br>The authors had adequately address mine and the other reviewers concerns. Whilst some may not be possible to be addressed, the authors had taken effort to improve the protocol such that I am now looking forward to a well rounded article after the study is conducted.
